# Discovering social learning ecosystems during clinical clerkship from United States medical students’ feedback encounters: a content analysis

**DOI:** 10.3352/jeehp.2024.21.5

**Published:** 2024-02-28

**Authors:** Anna Therese Cianciolo, Heeyoung Han, Lydia Anne Howes, Debra Lee Klamen, Sophia Matos

**Affiliations:** 1 Department of Medical Education, Southern Illinois University School of Medicine, Springfield, IL, USA; 2 Eccles Health Sciences Library, University of Utah, Salt Lake City, UT, USA; 3 Division of Surgery, Southern Illinois University School of Medicine, Springfield, IL, USA; Hallym University, Korea

**Keywords:** Ecosystem, Feedback, Medical students, Motivation, United States

## Abstract

**Purpose:**

We examined United States medical students’ self-reported feedback encounters during clerkship training to better understand in situ feedback practices. Specifically, we asked: Who do students receive feedback from, about what, when, where, and how do they use it? We explored whether curricular expectations for preceptors’ written commentary aligned with feedback as it occurs naturalistically in the workplace.

**Methods:**

This study occurred from July 2021 to February 2022 at Southern Illinois University School of Medicine. We used qualitative survey-based experience sampling to gather students’ accounts of their feedback encounters in 8 core specialties. We analyzed the who, what, when, where, and why of 267 feedback encounters reported by 11 clerkship students over 30 weeks. Code frequencies were mapped qualitatively to explore patterns in feedback encounters.

**Results:**

Clerkship feedback occurs in patterns apparently related to the nature of clinical work in each specialty. These patterns may be attributable to each specialty’s “social learning ecosystem”—the distinctive learning environment shaped by the social and material aspects of a given specialty’s work, which determine who preceptors are, what students do with preceptors, and what skills or attributes matter enough to preceptors to comment on.

**Conclusion:**

Comprehensive, standardized expectations for written feedback across specialties conflict with the reality of workplace-based learning. Preceptors may be better able—and more motivated—to document student performance that occurs as a natural part of everyday work. Nurturing social learning ecosystems could facilitate workplace-based learning such that, across specialties, students acquire a comprehensive clinical skillset appropriate for graduation.

## Graphical abstract

[Fig f3-jeehp-21-05]

## Introduction

### Background/rationale

Written commentary from clinical teachers is an important component of clinical performance assessment; rich narratives of medical student performance serve 2 roles: assessment for learning—facilitating reflection and goal-setting—and assessment of learning—supporting evaluation [1,2]. However, 2 limitations threaten written commentary’s utility: poor quality and low credibility. Due to social and material aspects of the learning environment [1], written comments can be sparse, untimely, generic, and overwhelmingly positive [2-4]. Moreover, written commentary can have less impact on competency judgments than quantitative metrics [5]. Environmental factors that negatively impact clinical teaching and feedback practices are not unique to Western countries; they have also been observed in Asia, where hierarchical and collectivist culture may also play a role [6,7].

Our school tried to address these factors in our undergraduate clerkship curriculum via reforms aimed at promoting clinical immersion and professional socialization [8]. The goal was to facilitate longitudinal teacher-learner relationships, thereby increasing students’ participation in clinical work and improving feedback quality. Reforms included the creation of “On the Fly” (OTF) forms requiring preceptors to document weekly their formative feedback observations regarding 8 medical student skills and attributes (oral case presentations, history-taking, physical examination, clinical reasoning, procedures/skills, patient notes). The forms’ electronic reporting system allowed students immediate feedback access to adjust their performance before rotation’s end. Clerkship grading committees used collated OTF commentary to make student progress decisions.

Despite early success in facilitating teacher-learner relationships and participation in clinical work [8,9], we found that OTF feedback shared the same limitations as written commentary generally. Students informally reported finding it unhelpful for learning, and neither students nor faculty believed it sufficient to make progress decisions other than pass/fail. Yet, our students claimed to receive quality feedback from their preceptors in situ. Hearing this prompted us to wonder: What does in situ feedback in our reformed curriculum look like?

### Objectives

We examined medical students’ self-reported feedback encounters during clerkship training to better understand in situ feedback practices. Specifically, we asked: Who do students receive feedback from, about what, when, where, and how do they use it? We explored whether curricular expectations for preceptors’ written commentary aligned with feedback as it occurs naturalistically in the workplace.

## Methods

### Ethics statement

This study was deemed non-human subjects research by the Springfield Committee on Human Subjects Research (protocol no., 21-850).

### Study design

We used a qualitative survey-based experience sampling approach [10], a feasible alternative to direct observation while coronavirus disease 2019 restrictions limited non-essential personnel in our clinical settings. This design also allowed students to describe their experiences in their own words. Given that learners’ perceptions can determine feedback’s impact [11], we thought their personal accounts should inform our effort.

### Setting

This study occurred from July 2021 to February 2022 at Southern Illinois University School of Medicine (SIUSOM), a small (class size=72), community-based medical school in the Midwestern United States. Clinical clerkships take place in Year 3 of the 4-year curriculum. Clerkship students rotate through 8 core specialties in a mix of hospital and clinic settings over an 8-month period (Table 1).

### Participants

We asked Year 1 and Year 2 curriculum coordinators to nominate students they found reflective and likely to share their learning experiences. To achieve maximum variation sampling, we considered these nominations together with class demographics and prior clinical performance. Among 18 students selected, we recruited 11 (61%) via personal email from the study’s principal investigator (A.T.C.). Participating students were 64% female, 27% from racial or ethnic groups historically marginalized in the United States, and 73% scoring in the middle quartiles on Year 2 clinical competency examinations. All participants received a US $25 Visa gift card for their participation.

### Variables

We analyzed the who, what, when, where, and why of students’ self-reported feedback encounters.

### Data source/measurement

Experience sampling consisted of an anonymous, weekly, 2-item qualitative survey (via SurveyMonkey; https://www.surveymonkey.com/) asking students to: (1) select their current clerkship; and (2) describe their most recent feedback experience, specifying who, what, when, where, and why (Supplement 1). Sampling occurred via automated email prompts every week of core clerkship instruction (i.e., 30 times) on a random weekday and time between 8:00 AM and 5:00 PM. Automated reminders were sent 24 hours later. Though we asked students to fill out every survey, we did not follow up with individuals who missed one. Two of the 11 participants deferred their first clerkship rotation, so the number of participants for the first 4 weeks of data collection was 9.

As the data were voluminous, yet relatively sparse in detail and contextualization, they were amenable to quantification but required a framework to guide analysis [12]. Therefore, we coded students’ survey responses using the 5Ws (Who, What, When, Where, Why) to organize our strategy. The first author (A.T.C.) reviewed the first 2 weeks of data to generate an initial codebook. The analysis team then analyzed the same data collaboratively, adding new codes and revising existing ones (Supplement 2). Team members coded the remaining data independently, meeting periodically to resolve coding questions or inconsistencies. When coding was finished, the first author (A.T.C.) summarized the code frequencies in tabular format (Supplement 3) and the team qualitatively mapped the frequency data to explore patterns in students’ feedback encounters. Raw response data are available in Dataset 1 and the coding data are available in Dataset 2.

### Bias mitigation/quality assurance

Our analysis team comprised 3 physicians (psychiatry, surgery, and pediatrics), 2 social sciences-trained PhD educators, 1 surgical nurse educator, and 1 medical librarian. All analysis team members identified as female, 6 had extensive practical or research experience with SIUSOM’s clerkship curriculum, and 2 identified as members of an ethnic group historically marginalized in the United States. We brought our diverse backgrounds to our data analysis to enrich our understanding of students’ feedback encounters, deferring to physicians’ perspectives when students’ accounts were clinically ambiguous. To member check our findings, we presented them to our participants for review and comment. We also gathered information about participants’ experiences with the survey to characterize their response process and identify threats to validity.

### Study size

We used maximum variation sampling to recruit a demographically and academically diverse participant sample comprising 15% of the class. We sampled participants’ experiences every week of the core clerkships.

### Statistical methods

We used descriptive statistics to present the results.

## Results

### Data summary

The average weekly response rate was high (85%). Students took 1 minute to 7 days to respond to the survey prompt, with the vast majority responding within 1–2 days. After removing 18 outliers where the student stepped away from the survey for several hours (a behavior confirmed via member checking), average survey completion time was 2 minutes. The data analysis team coded 272 responses out of a possible 322 (84%) if every student had filled out every survey, and 267 of these responses described feedback encounters. Average response length was 65 words (range, 5–205 words). Very short responses occurred rarely, such as when students reported having not received feedback. The frequency of reported feedback encounters reflected clerkship duration (Table 1).

### Students’ feedback encounters (Who, What, When, and Where)

**Fig. 1.**
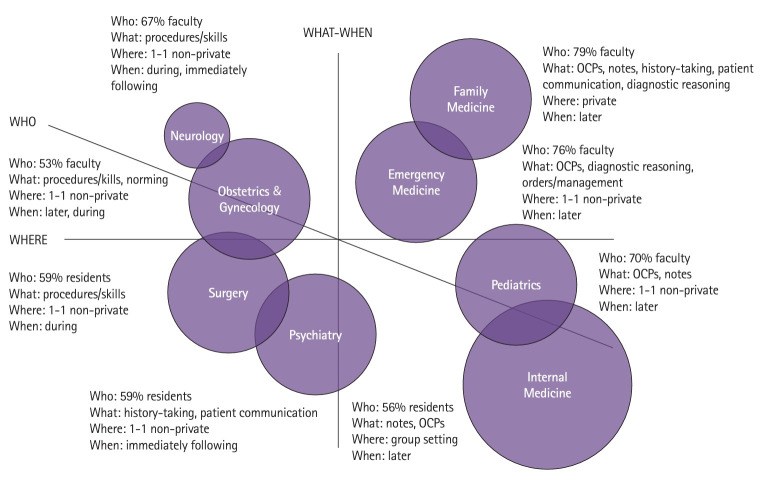
Qualitative map of the who, what, when, and where characteristics of United States medical students’ reported clinical feedback encounters from July 2021 to February 2022. OCPs, oral case presentations.

Fig. 1 presents a qualitative map of the code frequency data (Supplement 3). Each clerkship is shown as a circle, whose relative size represents the number of feedback encounters reported. The clerkships are arranged relative to 3 axes (What-When, Where, and Who) (Table 2) and to each other to reflect patterns among the frequencies.

Arranged along the What-When axis are clerkships in which feedback tended to target procedures or technical skills (e.g., suturing) and occurred during task performance (leftmost from the axis), targeted general clinical skills (e.g., oral case presentations, patient notes) and occurred after task performance (rightmost from the axis), or targeted clinical skills more characteristic of some specialties versus others (e.g., diagnostic reasoning, closer to the axis). Arranged along the Where axis are clerkships for which feedback tended to occur in private (highest above the axis), in non-private settings, such as the operating room (closer to the axis), or in groups, such as ward rounds (lowest below the axis). Arranged along the Who axis are clerkships for which feedback tended to come from faculty (above the axis), from residents (below the axis), or a balance of both (near the axis).

This map suggests that feedback occurred in patterns related to each core specialty’s work. For example, feedback in surgery tended to occur in the operating room, at procedure’s end, while the student sutured under a resident’s supervision. Feedback in neurology and obstetrics & gynecology similarly emphasized procedures and skills and was received at the bedside. In psychiatry, where rapport-building is a defining characteristic of patient care, students reported receiving feedback on patient communication more than twice as often as other clerkships. This feedback typically came from residents in non-private settings (e.g., the hallway) immediately following patient encounters. In family medicine, emergency medicine, pediatrics, and internal medicine, feedback tended to emphasize oral case presentations and notes, and the location of feedback encounters varied with the clinical setting. In family medicine, where students worked with a single faculty preceptor for 4 weeks, usually in a rural clinic without postgraduate trainees, feedback tended to occur privately (e.g., the faculty’s office). In internal medicine, where students spent most of their time in the hospital, oral case presentations—and feedback upon them—typically occurred on ward rounds as part of the service team’s work. Feedback in emergency medicine and family medicine, the specialties that see undifferentiated patients needing a diagnosis, tended to emphasize diagnostic reasoning, where other specialties did not.

### Students’ feedback encounters (Why)

**Fig. 2.**
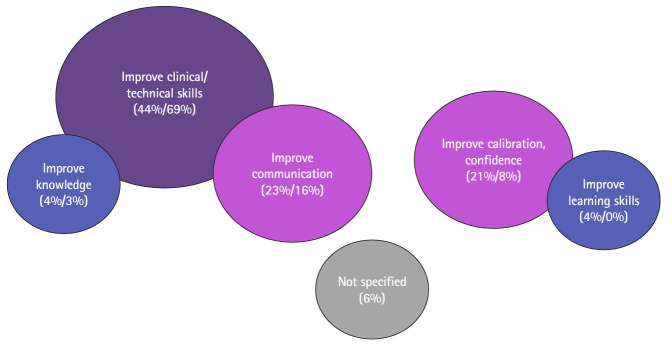
Why frequency characteristics of United States medical students’ reported clinical feedback encounters from July 2021 to February 2022.

In general, students reported using feedback to improve on the feedback’s topic. The majority of feedback topics related to technical/clinical skills, and students reported using feedback to improve these skills most often (Fig. 2, Supplement 3). Similarly, students reported receiving feedback on their knowledge in 3% of encounters and using feedback for knowledge development in 4%.

There was some variation too. Students received feedback on technical/clinical skills in 69% of encounters, but used feedback to improve these skills in 44%. Students reported using feedback to improve communication in 23% of encounters, but only 16% of feedback topics were coded as communication. Similarly, students reported using 21% of feedback encounters to calibrate their self-assessment and improve their confidence, though only 8% of reported feedback encounters addressed students’ overall performance. Students reported using 4% of feedback encounters to improve their learning skills, although no such feedback was coded. Notably, in 6% of feedback encounters, students did not report what utility the feedback had for their learning. They sometimes described the feedback they received as too generic to be called feedback.

## Discussion

### Key results

We investigated the who, what, when, where, and why characteristics of medical students’ self-reported feedback encounters in a clerkship curriculum reformed to facilitate clinical immersion and professional socialization. We identified patterns among our students’ experiences that seemed related to the nature of each core specialty’s work.

### Interpretation

Core clerkships appear to represent different “social learning ecosystems”—our term for the distinctive learning environments shaped by the social and material aspects of a given specialty’s work. Social learning ecosystems determine who preceptors are, what students do with preceptors, and what skills or attributes matter enough to preceptors to comment on. In these ecosystems, preceptors incorporate students into clinical work where they can do so safely and efficiently, and their feedback reflects effort to help students engage productively in this work.

These findings suggest that comprehensive, standardized expectations for written feedback across specialties conflict with the reality of workplace-based learning. Rather than exercising all 8 of our targeted physician skills and attributes equally in all specialties, our clerkship students appear to exercise samples of skills and attributes important to each specialty. Preceptors may be better able—and more motivated—to write feedback on these samples because engaging with them is a natural part of their everyday work. Developing contextualized samples of skills and attributes within rotations should, across rotations, amount to a comprehensive skillset appropriate for graduation as a junior physician.

### Comparison with previous studies

Consistent with theoretical frameworks of the learning environment [1], our results reinforce the idea that material and social context shapes students’ opportunities to participate in clinical work and therefore their feedback encounters. Our findings indirectly validate claims that our clerkship students participate in clinical work [8,9]. Social learning ecosystems may be considered a logical representation of the clinical workplace curriculum [13].

### Limitations

Our data quality depends on students’ accurate recall of their feedback encounters. Although students filled out the surveys in timely fashion, we cannot evaluate whether their recollections were the most recent or were salient for another reason. Also, our sample comprised students only. Direct observation of clinical teaching could have produced a different data set—one inclusive of preceptor behaviors—that would better inform OTF form redesign. Moreover, direct observation would have allowed sole focus on high-quality feedback encounters, which might produce a different dataset. Our study took place at a single US medical school with a pioneering pass/fail clerkship curriculum. Although other US medical schools have followed suit [14], and institutions worldwide are experimenting with clinical curriculum reform [7,15], educational philosophies predominant in traditional clerkship curricula or in other health care systems or national cultures may foster different social learning ecosystems [16].

### Suggestions

We used our findings to simplify the OTF form. Although it still invites preceptors to comment on the same 8 skills and attributes, we reduced the number of text fields from 8 to 1. We also reworded the feedback prompt to stimulate preceptors’ recall of their interactions with the student “as a junior member of the care team” and the feedback they gave in situ. We aim to help preceptors remain open regarding what they write feedback on, yet facilitate more detailed recall of interacting with students as part of clinical work. Further investigation should determine whether these changes improve OTF feedback utility and whether specialty-specific forms are indicated.

### Conclusion

We discovered patterns in clerkship students’ feedback encounters that may be attributable to specialty-specific social learning ecosystems. Nurturing these ecosystems could facilitate workplace-based learning such that, by the end of the curriculum, students have acquired a comprehensive clinical skillset.

## Figures and Tables

**Figure f3-jeehp-21-05:**
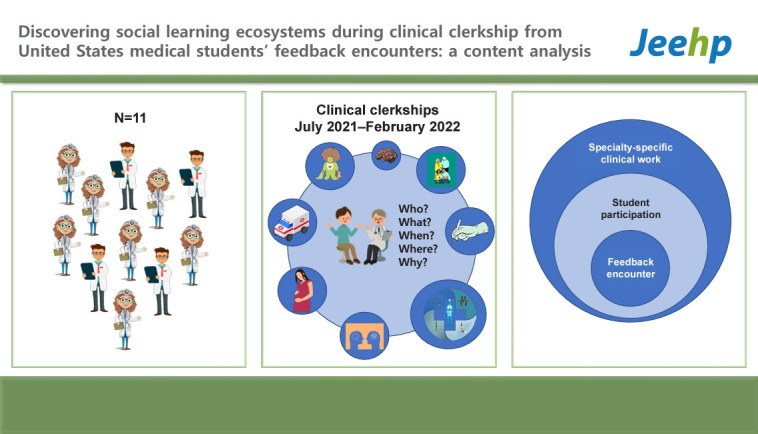


**Table 1.** Core clerkships at Southern Illinois University School of Medicine—specialty, duration, setting, and number of reported feedback encounters

Specialty	Duration	Setting	No. of reported feedback encounters
Internal Medicine	4 weeks/2 weeks	Hospital/clinic	56
Psychiatry	2 weeks/2 weeks	Hospital/clinic	37
Emergency Medicine	4 weeks	Hospital	35
Family Medicine	4 weeks	Clinic	33
Surgery	4 weeks	Hospital with clinic days	33
Pediatrics	2 weeks/2 weeks	Hospital/clinic	30
Obstetrics/Gynecology	4 weeks	Hospital with clinic days	29
Neurology	2 weeks	Clinic	14

**Table 2.** Qualitative map axis description

Name	Orientation	Description
What-When (feedback content and timing)	Vertical	Procedures/technical skills vs. general clinical skills vs. specialty clinical Skills
		Later vs. during vs. immediately following
Where (feedback location)	Horizontal	Private vs. 1-on-1 non-private vs. group setting
Who (feedback source)	Diagonal	Faculty vs. residents

## Footnotes

**Data availability**
Data files are available from Harvard Dataverse: https://doi.org/10.7910/DVN/YMDOBE Dataset 1. Raw data of United States medical students’ reported clinical feedback encounters from July 2021 to February 2022.

Dataset 2. Coding data of United States medical students’ reported clinical feedback encounters from July 2021 to February 2022.

## Supplementary materials

Supplementary files are available from Harvard Dataverse: https://doi.org/10.7910/DVN/YMDOBE

Supplement 1. Survey form of United States medical students’ feedback during their clinical clerkship from July 2021 to February 2022.

Supplement 2. Survey response codebook of United States medical students’ feedback during their clinical clerkship from July 2021 to February 2022.

Supplement 3. Who, What, When, Where, & Why code frequency data in tabular format of United States medical students’ feedback during their clinical clerkship from July 2021 to February 2022.

Supplement 4. Audio recording of the abstract.
