# Supplementary material for: Discovering social learning ecosystems during clinical clerkship from United States medical students’ feedback encounters: a content analysis
Source: J Educ Eval Health Prof. 2024 Feb 28;21:5. doi: 10.3352/jeehp.2024.21.5 (PMC10948917; doi:10.3352/jeehp.2024.21.5)
Supplement: Supplementary file 3 — Supplement 1. Survey form of United States medical students’ feedback during their clinical clerkship from July 2021 to February 2022. [file jeehp-21-05-suppl1.docx]

**Supplement 1.** Survey form

**Clerkship feedback experiences survey**

1. What rotation are you on? (select from pulldown menu)

• Emergency Medicine

• Family Medicine

• Internal Medicine

• Neurology

• Obstetrics and Gynecology

• Pediatrics

• Psychiatry

• Surgery

2. Feedback may be defined as information a learner uses to make adjustments in pursuit of a goal [1].

Please describe your most recent feedback experience on this rotation. Please specify the feedback source, what the feedback was about, how the feedback occurred, and under what conditions. Please provide any additional detail as you see fit to thoroughly and accurately describe your experience.

To complete this task, please specifically identify who the feedback came from (e.g., preceptor, resident, nurse educator, peer, patient); what the content of the feedback was; when you received it (e.g., time of day, before/during/after a specific event); where you received feedback (e.g., location, one-on-one or in front of a group); why this feedback relates to your development as a physician.

(blank text field provided)

**Reference**

1. Ende J. Feedback in clinical medical education. JAMA 1983;250:777-781. <https://doi.org/10.1001/jama.1983.03340060055026>
