# Supplementary material for: Discovering social learning ecosystems during clinical clerkship from United States medical students’ feedback encounters: a content analysis
Source: J Educ Eval Health Prof. 2024 Feb 28;21:5. doi: 10.3352/jeehp.2024.21.5 (PMC10948917; doi:10.3352/jeehp.2024.21.5)
Supplement: Supplementary file 4 — Supplement 2. Survey response codebook of United States medical students’ feedback during their clinical clerkship from July 2021 to February 2022. [file jeehp-21-05-suppl2.docx]

**Supplement 2.** Survey response codebook

Each survey response constitutes a single unit of data such that multiple codes may be applied to the same survey response.

**Who—**Please code who the student specifies as the feedback provider as follows:

**• Clinical faculty:** may be specified as an attending, a preceptor, a faculty member, a faculty supervisor, or by name; non-faculty supervising physicians (i.e., community preceptors of family medicine rotations) also fall into this category, as distinct from trainees (see Medical Trainee below)

**• Other faculty:** non-clinical faculty; may be specified as basic science faculty, medical education faculty, research faculty, or by name; also includes clerkship advisor, who may be clinical faculty, but does not provide feedback in the clinical setting

**• Medical trainee:** may be specified as a resident, chief resident, or fellow

**• Nurse educator:** specified as “nurse educator” or by name

**• Other clinician:** may be a nurse, physician assistant, surgical tech, or any other non-physician care provider who is not a nurse educator (see above)

**• Patient:** this may be specified as “patient” or by name; may include patient family member or caregiver

**• Peer:** may be specified as a peer, “MS*” (i.e., medical student, any year), classmate, etc.; only medical students fall in this code (see “other trainee” below)

**• Other trainee:** this feedback provider is a non-physician trainee, such as a nursing or physician assistant student

**• Other:** includes people not listed above, such as research coordinators or people from the students’ non-medical social circles (e.g., family members, church leaders, etc.); also includes instances where the provider cannot be inferred (e.g., student reports getting feedback from the “On the Fly” form but does not specify who filled it out; student gains feedback from reviewing the electronic health record and that feedback is not attributable to anyone) (please specify)

**What—**Please code what the student reports the feedback to be about as follows:

**• History taking:** may include “interviewing,” depending on context (i.e., whether the response foregrounds information gathering over patient interactions, such as building rapport, asking open-ended questions, listening empathically, etc.)

**• Physical examination:** including neurological examination; when the response says “H&P” (History & Physical), please include this code along with History Taking

**• OCPs (****oral case presentations)**

**• Notes**

**• Patient Communication skills:** may include “interviewing,” depending on context (i.e., whether the response foregrounds patient interactions such as building rapport, asking open-ended questions, listening empathically, etc. over information gathering); includes communication with family and/or caregivers

**• Teamwork:** aspects of team performance, e.g., dependability, reliability, being prepared, and communication with team members

**• Knowledge base**

**• Diagnostic reasoning**

**• Orders/management**

**• Procedures/skills:** includes, e.g., suturing, catching babies, etc. (please specify in comments)

**• Norming:** feedback on how the student is doing in the clerkship overall, compared to other students, relative to expectations, etc.

**• Other:** feedback content not specified above (please specify)

**When –** Please code when the student reports the feedback was provided as follows:

- **During –** feedback is reported as being provided while the activity was ongoing
- **Immediately following –** feedback is reported as being provided immediately after an activity was completed
- **Later –** feedback is reported as occurring after an activity, but how long after is not specified
- **Feedback conversation** – an occurrence of formal or summarizing feedback conversation is reported, such as when an On the Fly form is filled out, at the mid-rotation evaluation, during a clerkship advising meeting, etc.
- **NA** – timing of feedback is not discernable from the response

**Where –** Please code where the student reports receiving feedback as follows:

- **One on one** –a student explicitly mentions that feedback was one on one; one on one may also be inferred from the nature of feedback provided (e.g., preceptor offers a detailed explanation for medication use, how to do a procedure, etc.)
  - **Private** – add this code to “One on one” if the student specifies that feedback was given privately
  - **Non-private** – add this code to “One on one” if the student specifies that they were in a public setting (e.g., hallway, operating room, emergency department pod) or that others were around, even if these others weren’t participating in the feedback encounter
  - *NOTE – Do not add a sub-code to “One on one” if the student does not specify the feedback setting*
- **Group setting** – the student reports that the feedback was given as part of a group activity, such as rounds, classroom teaching, etc.
- **OTF** – the student specifies that feedback was provided on the On the Fly form (this code may be added to another “Where” code)
- **NA** – student does not specify at all where the feedback is given

**Why –** Please code why the student reports the feedback relates to their development as a physician as follows:

- **Improve clinical/technical skills**
- **Improve communication with patients and families/caregivers**
- **Improve knowledge base**
- **Improve professionalism/teamwork skills/communication with team members**
- **Improve learning skills**
- **Improve calibration/confidence –** the student notes feeling reassured about where they stand in the clerkship or with the clinical team, expresses greater confidence in their self-assessment
- **NA –** why the feedback contributes to development is not specified
