# Supplementary material for: Discovering social learning ecosystems during clinical clerkship from United States medical students’ feedback encounters: a content analysis
Source: J Educ Eval Health Prof. 2024 Feb 28;21:5. doi: 10.3352/jeehp.2024.21.5 (PMC10948917; doi:10.3352/jeehp.2024.21.5)
Supplement: Supplementary file 5 — Supplement 3. Who, What, When, Where, & Why code frequency data in tabular format of United States medical students’ feedback during their clinical clerkship from July 2021 to February 2022. [file jeehp-21-05-suppl3.docx]

**Supplement 3.** Who, What, When, Where, & Why code frequency data in tabular format

**Table 1.** Who frequency data

| Rotation | Clinical faculty | Medical trainee | Other clinician | Patient | Peer | Source not specified | Total |
| --- | --- | --- | --- | --- | --- | --- | --- |
| EM^a)^ | **76 (28)** | 24 (9) | - | - | - |  | 100 (37) |
| FM | **79 (26)** | 18 (6) | - | 3 (1) | - |  | 100 (33) |
| IM^a)^ | 43 (26) | **56 (34)** | - | 2 (1) | - |  | 100 (61) |
| Neurology^a)^ | **67 (10)** | 7 (1) | 20 (3) | 7 (1) | - |  | 100 (15) |
| OB/GYN^a)^ | **53 (16)** | 47 (14) | - | - | - |  | 100 (30) |
| Peds | **70 (21)** | 20 (6) | 3 (1) | - | 3 (1) | 3 (1) | 100 (30) |
| Psych^a)^ | 33 (13) | **59 (23)** | 5 (2) | - | 3 (1) | - | 100 (39) |
| Surgery^a)^ | 32 (11) | **59 (20)** | 6 (2) | 3 (1) | - | - | 100 (34) |
| Total | **54 (151)** | 41 (113) | 3 (8) | 1 (4) | <1 (2) | <1 (1) | 100 (279) |

Values are presented as % (number). Bolded values represent the most frequently reported feedback source in each rotation.

EM, Emergency Medicine; FM, Family Medicine; IM, Internal Medicine; OB/GYN, Obstetrics & Gynecology; Peds, Pediatrics; Psych, Psychiatry.

^a)^At least one survey response for this specialty described a feedback encounter involving more than one feedback source.

Table 2. What Frequency Data

| Feedback Topic | Rotation | | | | | | | | |
| --- | --- | --- | --- | --- | --- | --- | --- | --- | --- |
|  | EM | FM | IM | Neurology | OB/GYN | Peds | Psych | Surgery | TOTAL |
| History-Taking | 8% (5) | 13% (7) | 8% (6) | 6%  (1) | 5%  (2) | 7%  (3) | **30%**  **(16)** | 12%  (5) | 12%  (45) |
| Physical Examination | 3% (2) | 9% (5) | 4% (3) | **41%**  **(7)** | 11%  (4) | 7%  (3) | - | 7%  (3) | 7%  (27) |
| Oral Case Presentations | **28% (17)** | 21% (11) | **32% (24)** | 12%  (2) | 8%  (3) | 26%  (11) | 6%  (3) | 7%  (3) | 19%  (74) |
| Notes | 2% (1) | 19% (10) | **30% (22)** | 6%  (1) | 8%  (3) | 19%  (8) | 17%  (9) | 14%  (6) | 16%  (60) |
| Patient Communication | 8% (5) | 11% (6) | 8% (6) | 18%  (3) | 8%  (3) | 12%  (5) | **33%**  **(18)** | 10%  (4) | 13%  (50) |
| Teamwork | 2% (1) | - | 5% (4) | - | 5%  (2) | 2%  (1) | 2%  (1) | 7%  (3) | 3%  (12) |
| Knowledge | 3% (2) | 4% (2) | - | - | 5%  (2) | 9%  (4) | 2%  (1) | 5%  (2) | 3%  (13) |
| Clinical Reasoning | **23% (14)** | 9% (5) | 1% (1) | 6%  (1) | - | 5%  (2) | 2%  (1) | - | 6%  (24) |
| Orders/ Management | 10% (6) | 2% (1) | 1% (1) | - | - | - | - | - | 2%  (8) |
| Procedures/ Skills | 5% (3) | - | - | - | **26%**  **(10)** | 5%  (2) | - | **26%**  **(11)** | 7%  (26) |
| Norming | 5% (3) | 6% (3) | 7% (5) | 12%  (2) | 16%  (6) | 7%  (3) | 9%  (5) | 10%  (4) | 8%  (31) |
| Other | 2% (1) | 6% (3) | 3% (2) | - | 8%  (3) | 2%  (1) | - | 5%  (2) | 3%  (12) |
| TOTAL | 100%  (60) | 100%  (53) | 100%  (74) | 100%  (17) | 100%  (38) | 100%  (43) | 100%  (54) | 100%  (43) | 100%  (382) |

EM = Emergency Medicine, FM = Family Medicine, IM = Internal Medicine, OB/GYN = Obstetrics & Gynecology, Peds = Pediatrics, Psych = Psychiatry. **Bolded** values represent the clear majority of feedback topics reported for a given rotation. These values are typically proportions of 25% or higher and approximately 10 percentage points higher than the next-highest proportion. Underlined values represent proportions of feedback topics that collectively represent the clear majority of feedback topics reported for a given rotation. Summed, these values represent more than 50% of reported feedback topics, but they do not individually stand out as a clear majority (see FM and Peds).

Table 3. When Frequency Data

| Feedback Timing | Rotation | | | | | | | | |
| --- | --- | --- | --- | --- | --- | --- | --- | --- | --- |
|  | EM* | FM* | IM | Neurology* | OB/GYN* | Peds* | Psych* | Surgery | TOTAL |
| During | 8%  (3) | 3%  (1) | 7%  (4) | **25%**  **(4)** | **25%**  **(8)** | 13%  (4) | 11%  (4) | **33%**  **(11)** | 14%  (39) |
| Immediately Following | 17%  (6) | 24%  (8) | **38%**  **(21)** | 25%  (4) | 13%  (4) | 26%  (8) | **42%**  **(16)** | 30%  (10) | 28%  (77) |
| Later | **56%**  **(20)** | 41%  (14) | 32%  (18) | 31%  (5) | 44%  (14) | 45%  (14) | 32%  (12) | 15%  (5) | 37%  (102) |
| Feedback Conversation | 11%  (4) | 21%  (7) | 16%  (9) | 13%  (2) | 19%  (6) | 13%  (4) | 11%  (4) | 15%  (5) | 15%  (41) |
| Timing Not Discernable | 8%  (3) | 12%  (4) | 7%  (4) | 6%  (1) | - | 3%  (1) | 5%  (2) | 6%  (2) | 6%  (17) |
| TOTAL | 100%  (36) | 100%  (34) | 100%  (56) | 100%  (16) | 100%  (32) | 100%  (31) | 100%  (38) | 100%  (33) | 100%  (276) |

^*^This rotation had at least one survey response in which multiple feedback experiences were described.

EM = Emergency Medicine, FM = Family Medicine, IM = Internal Medicine, OB/GYN = Obstetrics & Gynecology, Peds = Pediatrics, Psych = Psychiatry. **Bolded** values represent the clear majority of feedback timing reported across rotations. These values are proportions of 25% or higher and approximately 10% higher than the next highest proportion. Across rotations, the frequency of reported feedback conversations or feedback timing that could not be discerned did not appear notably different.

Table 4. Where Frequency Data

| Feedback Location | Rotation | | | | | | | | |
| --- | --- | --- | --- | --- | --- | --- | --- | --- | --- |
|  | EM | FM | IM | Neuro | OB/GYN* | Peds | Psych* | Surgery* | TOTAL |
| One-on-One | 29%  (10) | 21% (7) | 23% (13) | 29%  (4) | 19%  (6) | 23%  (7) | 16%  (6) | 18%  (6) | 22%  (59) |
| One-on-One (private) | 3%  (1) | **33% (11)** | 9%  (5) | 7%  (1) | 9%  (3) | 17%  (5) | 16%  (6) | 6%  (2) | 13%  (34) |
| One-on-One  (non-private) | 43%  (15) | 27%  (9) | 21% (12) | 43%  (6) | 44%  (14) | 37%  (11) | 37%  (14) | **56%**  **(19)** | 37%  (100) |
| Group | 6%  (2) | 3%  (1) | **36%**  **(20)** | 7%  (1) | 13%  (4) | 10%  (3) | 16%  (6) | 15%  (5) | 15%  (41) |
| On the Fly Form | 11%  (4) | 6%  (2) | 2%  (1) | 7%  (1) | 13%  (4) | 10%  (3) | 5%  (2) | - | 6%  (17) |
| Location Not Specified | 9%  (3) | 9%  (3) | 9%  (5) | 7%  (1) | 3%  (1) | 3%  (1) | 11%  (4) | 6%  (2) | 7%  (20) |
| TOTAL | 100% (35) | 100% (33) | 100% (56) | 100% (14) | 100%  (32) | 100% (30) | 100% (38) | 100% (34) | 100%  (272) |

*At least one survey response for this rotation received multiple codes for When.

EM = Emergency Medicine, FM = Family Medicine, IM = Internal Medicine, Neuro = Neurology, OB/GYN = Obstetrics & Gynecology, Peds = Pediatrics, Psych = Psychiatry. **Bolded** values represent the clear majority of feedback locations reported across rotations. These values are proportions of 30% or higher and more than 10% higher than the next highest proportion. Across rotations, the frequency of reporting one-on-one feedback, feedback using the On the Fly form, or unspecified feedback location did not appear notably different.

Table 5. Why Frequency Data

| Feedback Use/Goal | Rotation | | | | | | | | |
| --- | --- | --- | --- | --- | --- | --- | --- | --- | --- |
|  | EM | FM | IM | Neurology | OB/GYN | Peds | Psych | Surgery | TOTAL |
| Clinical/ Technical Skills | **47%**  **(24)** | **51%**  **(21)** | **46%**  **(33)** | **58%**  **(11)** | **42%**  **(15)** | **40%**  **(18)** | 36%  (19) | 38% (15) | 44%  (156) |
| Patient Communication | 8%  (4) | 12%  (5) | 4%  (3) | 16%  (3) | 6%  (2) | 9%  (4) | 28%  (15) | 5%  (2) | 11%  (38) |
| Professionalism/ Teamwork/ Non-Patient Communication | 12%  (6) | 12%  (5) | 18%  (13) | - | 11%  (4) | 13%  (6) | 6%  (3) | 13%  (5) | 12%  (42) |
| Knowledge | 6%  (3) | 10%  (4) | - | - | 8%  (3) | 7%  (3) | 2%  (1) | 5%  (2) | 4%  (16) |
| Learning Skills | 2%  (1) | 2%  (1) | - | 5%  (1) | - | 7%  (3) | 2%  (1) | 8%  (3) | 3%  (10) |
| Calibration/ Confidence | 22%  (11) | 10%  (4) | 22%  (16) | 11%  (2) | 28%  (10) | 24%  (11) | 21%  (11) | 23%  (9) | 21%  (74) |
| Feedback Use/Goal Not Specified | 4%  (2) | 2%  (1) | 10%  (7) | 11%  (2) | 6%  (2) | - | 6%  (3) | 10%  (4) | 6%  (21) |
| TOTAL | 100% (51) | 100% (41) | 100% (72) | 100%  (19) | 100% (36) | 100% (45) | 100% (53) | 100% (40) | 100%  (357) |

EM = Emergency Medicine, FM = Family Medicine, IM = Internal Medicine, Neuro = Neurology, OB/GYN = Obstetrics & Gynecology, Peds = Pediatrics, Psych = Psychiatry. **Bolded** values represent the clear majority of feedback use/goal reported for a given rotation. These values are proportions of 40% or higher and at least 15 percentage points higher than the next highest proportion. Underlined values represent proportions of feedback use/goal that collectively represent the clear majority of feedback uses/goals reported for a given rotation. Summed, these values represent more than 60% of reported feedback topics, but they do not individually stand out as a clear majority (see Psych and Surgery).
